# Supplementary material for: Perineuronal nets stabilize the grid cell network
Source: Nat Commun. 2021 Jan 11;12:253. doi: 10.1038/s41467-020-20241-w (PMC7801665; doi:10.1038/s41467-020-20241-w)
Supplement: Supplementary file 1 — Supplementary information [file 41467_2020_20241_MOESM1_ESM.pdf]

Supplementary information

This section contains Supplementary Figure 1-14 and Supplementary tables 1-11.

# ANIMAL	GROUP	IMPLANT		NUMBER OF UNITS		NUMBER OF GRID CELLS	LFP
		R	L	R	L		
**1205**	Control	CA1	None	4	0		R
**1292**	Control	None	CA1	0	5		L
**1377**	Control	CA1	CA1	0	27		R+L
**1346**	Control	MEC	MEC	5	23	4	R+L
**1520**	chABC	MEC	MEC	0	92	11	L
**1522**	chABC	MEC	MEC	0	35	8	L
**1526**	chABC	MEC	MEC	5	47	9	R+L
**1528**	Control	MEC	MEC	97	0	36	R
**1529**	Control	MEC	MEC	36	11	9	R+L
**1531**	Control	CA1	CA1	0	11		R+L
**1588**	Control	MEC	CA1	0	1		L
**1589**	Control	MEC	CA1	0	4		L
**1626**	chABC	MEC	MEC	96	87	20	R+L
**1627**	chABC	MEC	MEC	0	84	22	L
**1677**	chABC	CA1	MEC	16	12	6	R+L
**1678**	chABC	None	MEC	0	9	3	L
**1679**	Control	None	MEC	0	0		L
**1688**	chABC	CA1	MEC	11	0		R
**1751**	Control	MEC	MEC	54	35	26	R+L
**1752**	Control	MEC	MEC	8	45	9	R+L
**1753**	Control	MEC	MEC	13	46	31	R+L
**1794**	chABC	CA1	CA1	14	10		R+L
**1795**	chABC	CA1	CA1	49	31		R+L
**TOTAL**	**Control**	**chABC**		**CA1**	**MEC**	**Grid cells**	
**23**	**13**	**10**		**185**	**840**	**194**	

Supplementary Table 1: Overview of animals used for electrophysiological recordings. R/L: Right/Left hemisphere

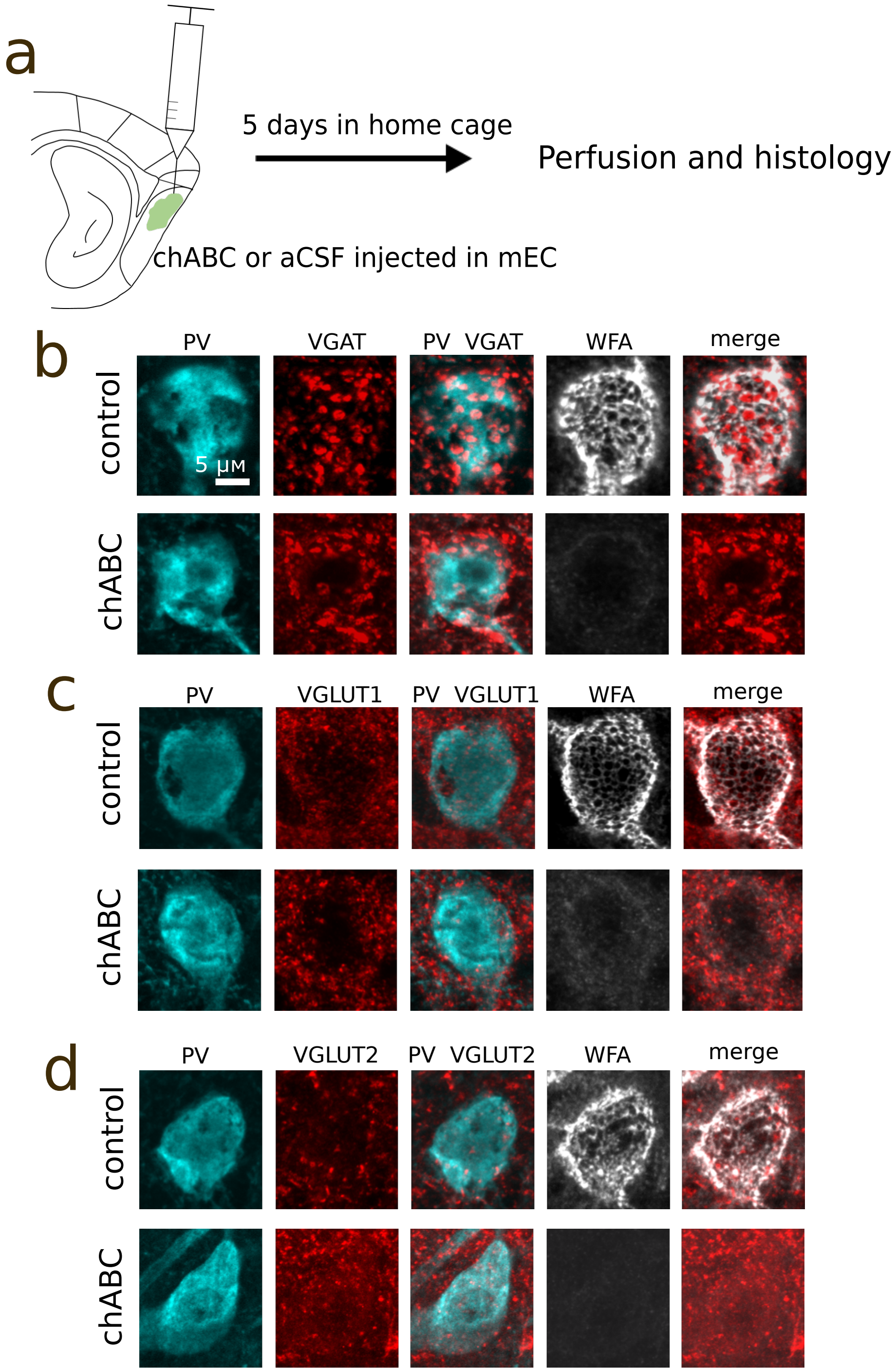

Supplementary Figure 1: Alterations of synaptic boutons on PV$^+$ cells after PNN removal. **a**) Experimental overview. chABC and aCSF was injected into opposite hemispheres of MEC in each animal. After five days in the home cage, animals were perfused and sections from both hemispheres stained and imaged in parallel for parvalbumin, WFA+ PNNs and presynaptic markers. **b-d**) Example images of single cells from aCSF and chABC treated hemispheres in MEC. Significant reduction in VGAT staining in the chABC treated hemispheres, indicating a reduction of GABAergic synapses onto PV$^+$ cells in MEC after PNN removal (quantification in main text Figure 1).

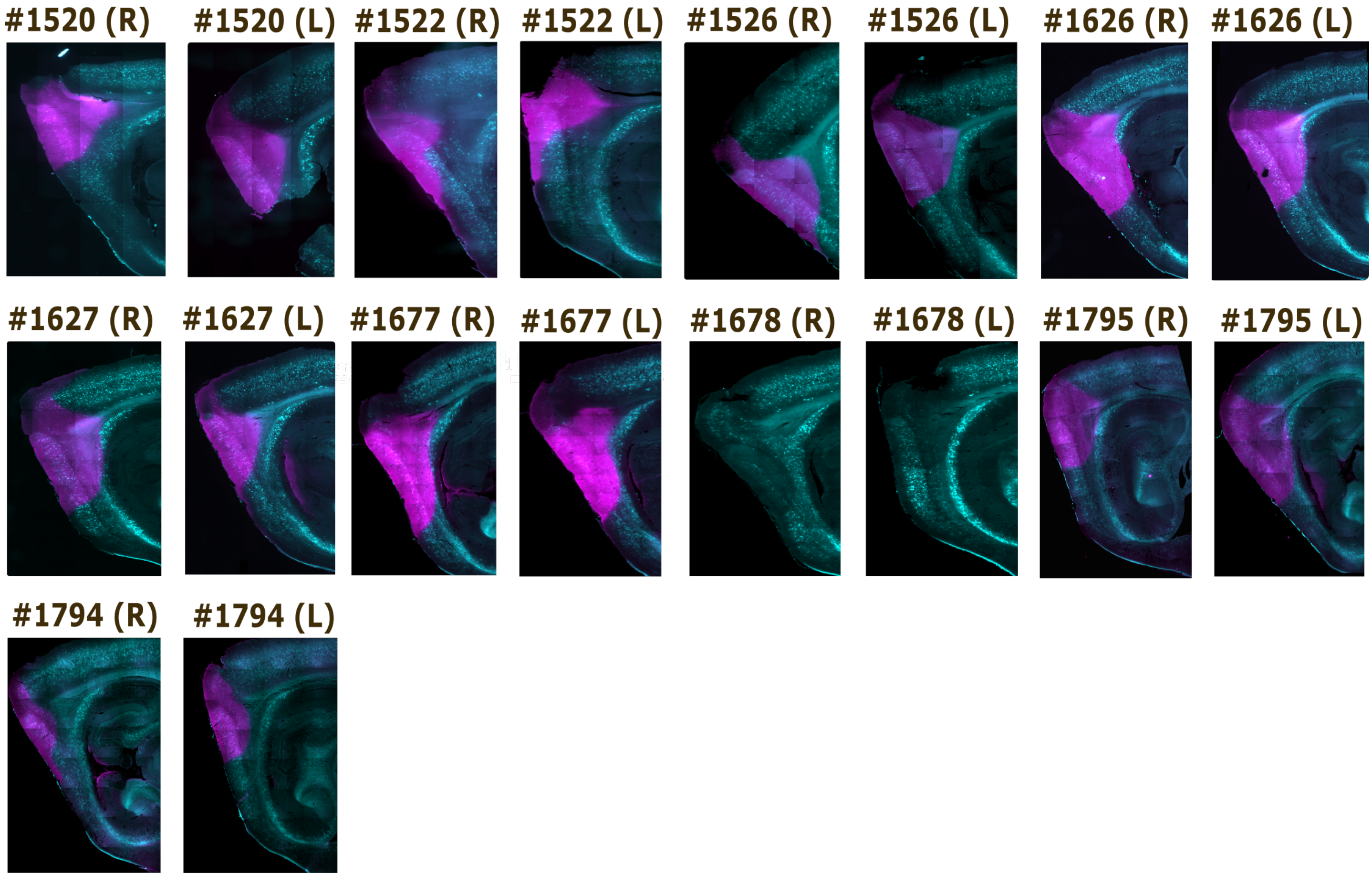

Supplementary Figure 2: Confirmed PNN removal in MEC of experimental animals. Sections were stained with WFA (cyan) and 3B3 (magenta) that labels CS6 "stubs" left after chABC degradation of chondroitin sulfate proteoglycans. Note that animal #1678 is missing the 3B3 staining but the chABC injection is still clearly visible due to low intensity WFA staining in the injected area.

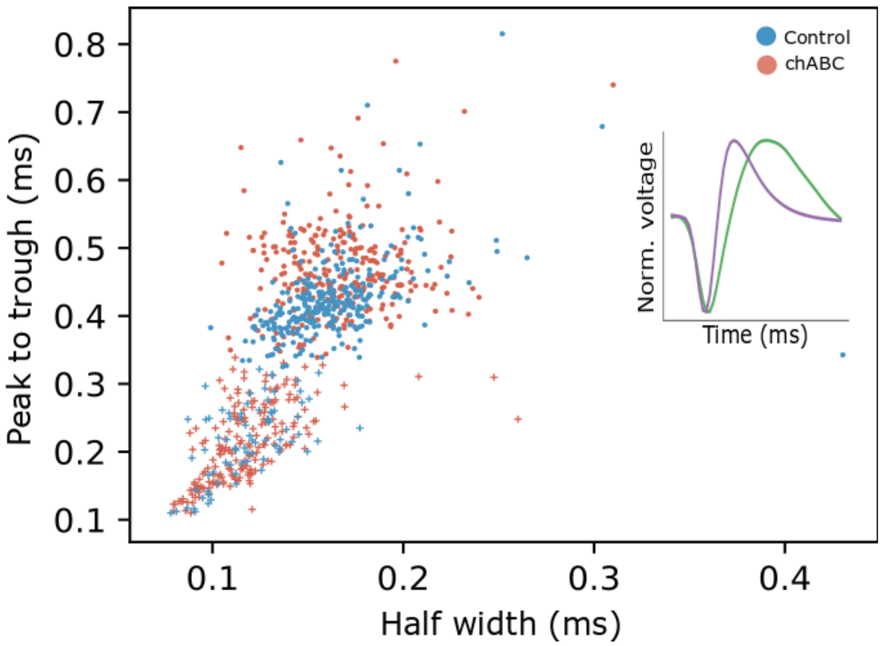

Supplementary Figure 3: Classification of broad and narrow spiking units. a) Units are separated into broad or narrow spiking based on peak to trough time and the half width of amplitude using kmeans. Circles represent units categorized as broad spiking and crosses represent units categorized as narrow spiking. Units from control animals in blue and chABC treated animals in red. Inset show examples of broad spiking (green) and narrow spiking units (purple).
b) Average firing rates: (mean $\pm$ sem)
Broad spiking; control 1.69 $\pm$ 0.09 Hz, chABC 1.40 $\pm$ 0.07 Hz ($U = 43784$, $p = 0.021$).
Narrow spiking; 5.55 $\pm$ 0.90 Hz, chABC 4.86 $\pm$ 0.62 Hz ($U = 8226$, $p = 0.045$).
Mann-Whitney U test (two-sided).

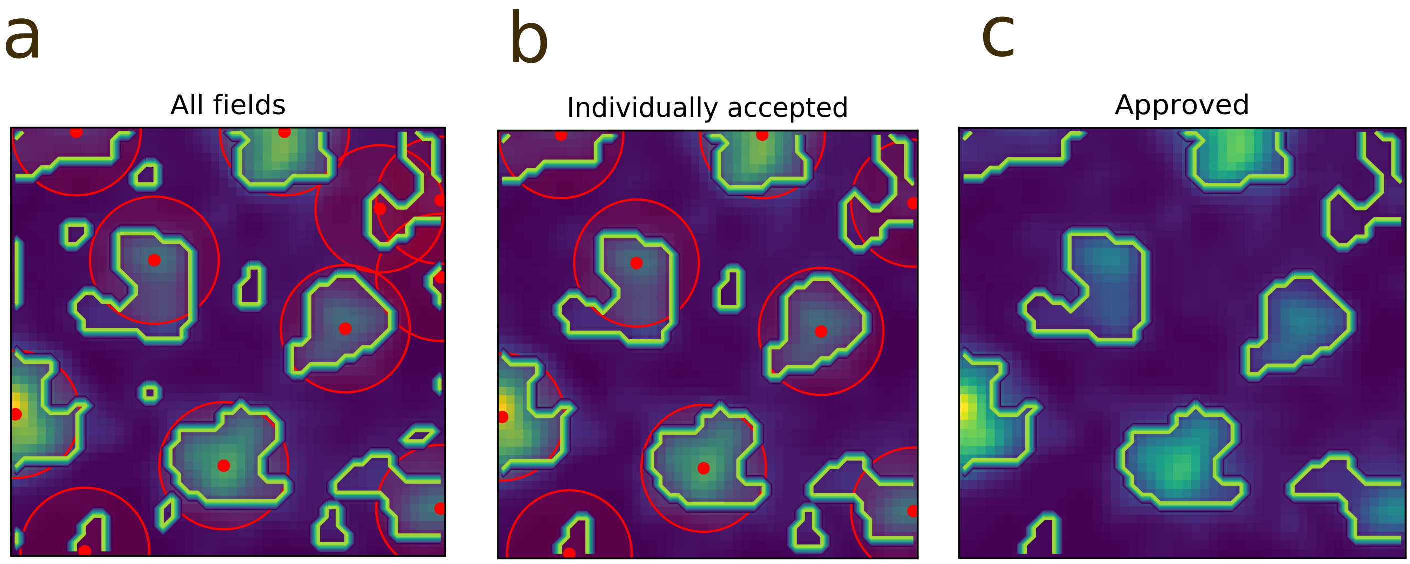

Supplementary Figure 4: Grid field detection. To estimate the firing within and outside fields, we first identified the individual fields in the rate map using a dual methods approach. **a**) First we defined the extent of each field, by calculating the Laplacian ∇^2 of the smoothed rate map to obtain its curvature (outlined by blue and yellow). Regions with a positive Laplacian, which are the valleys of the rate map, were excluded. To limit random out-of-field rate getting considered individual fields using the laplacian, we included the protocol of [**?**], where we calculated a global field radius using $0,7$ times half the distance from the center peak to the closest peak in the autocorrelogram (field center is marked by a red dot and the global field radius is marked by a red circle). **b**) Next, we excluded the lowest of any two peaks within a distance shorter than the global field radius along with any regions with an area less than 9 bins. **c**) Fields were defined to be any labeled region found after taking the Laplacian that corresponded with a non-excluded peak from the protocol of [**?**].

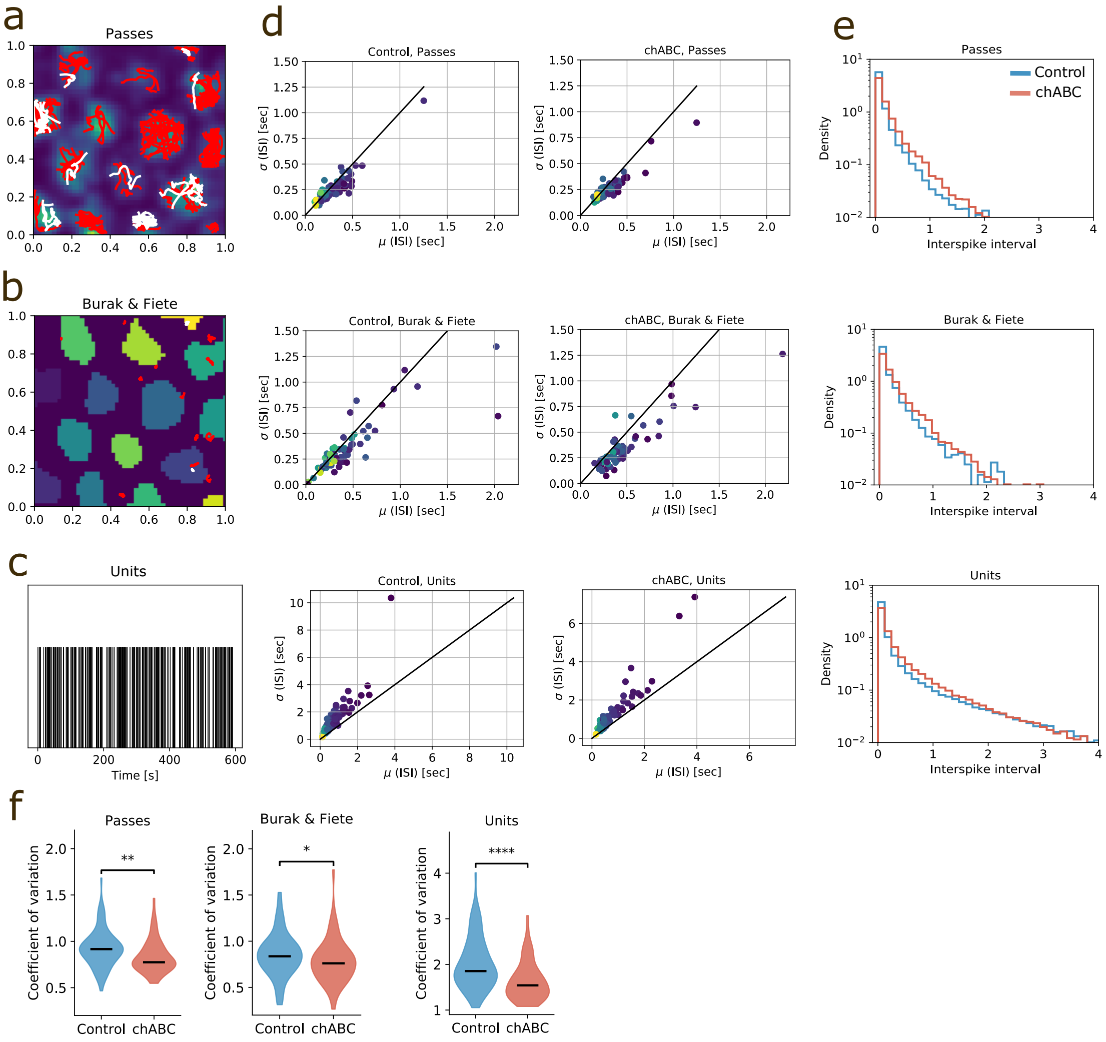

Supplementary Figure 5: Grid cell spike trains show reduced coefficient of variation (CV) of interspike intervals (ISI) after chABC treatment. Three different methods for calculating CV of ISI for grid cells in a familiar environment: **a**) ISI from passes through fields. Grid fields were identified as described in Supplementary Fig. 4. ISIs from all passes with duration longer than 1s and more than two spikes are used to calculate an ISI mean and standard deviation for the session. White lines mark accepted passes, discarded passes red lines. **b**) Speed-filtered ISI: Parts of the recording where the speed of the rat is lower than 8 cm/s, as described in [**?**]. ISIs from all parts of the recording with duration longer than 1s and more than two spikes are used to calculate an ISI mean and standard deviation for the session. White lines mark accepted and red discarded parts. Since the rats were mostly running above 10cm/s most of the data were discarded in the analysis. Thus, the method is not suitable for this kind of behavior. **c**) The unit method uses the spike train from one unit during the whole 10 min session to calculate its ISI mean and standard deviation. **d**) Standard deviation of the ISI σ versus mean ISI μ, for each of the three methods and both groups. Each data point is from a unique session, one session per unit. Groupwise CV is indicated by a least squares fit. Note the different axis limits due to larger values with the Unit method. **e**) Distribution of the interspike interval for the three methods. Deviation from a straight line indicates deviation from a poisson process. **f**) Distribution of coefficient of variation for all three methods and both groups. For all methods CV was lower in chABC treated animals. Passes: Control 0.99 vs chABC 0.82 , $p < 0.001$. Speed filtered: Control 0.84 vs chABC 0.77 , $p < 0.055$. Units: Control 1.84 vs chABC 1.54 , $p < 0.001$. Mann-Whitney U test (two-sided). $ns = notsignificant$, $^{*}p < 0.05$, $^{**}p < 0.01$, $^{***}p < 0.001$, $^{****}p < 0.0001$. Violin plots show min to max and median (black line).

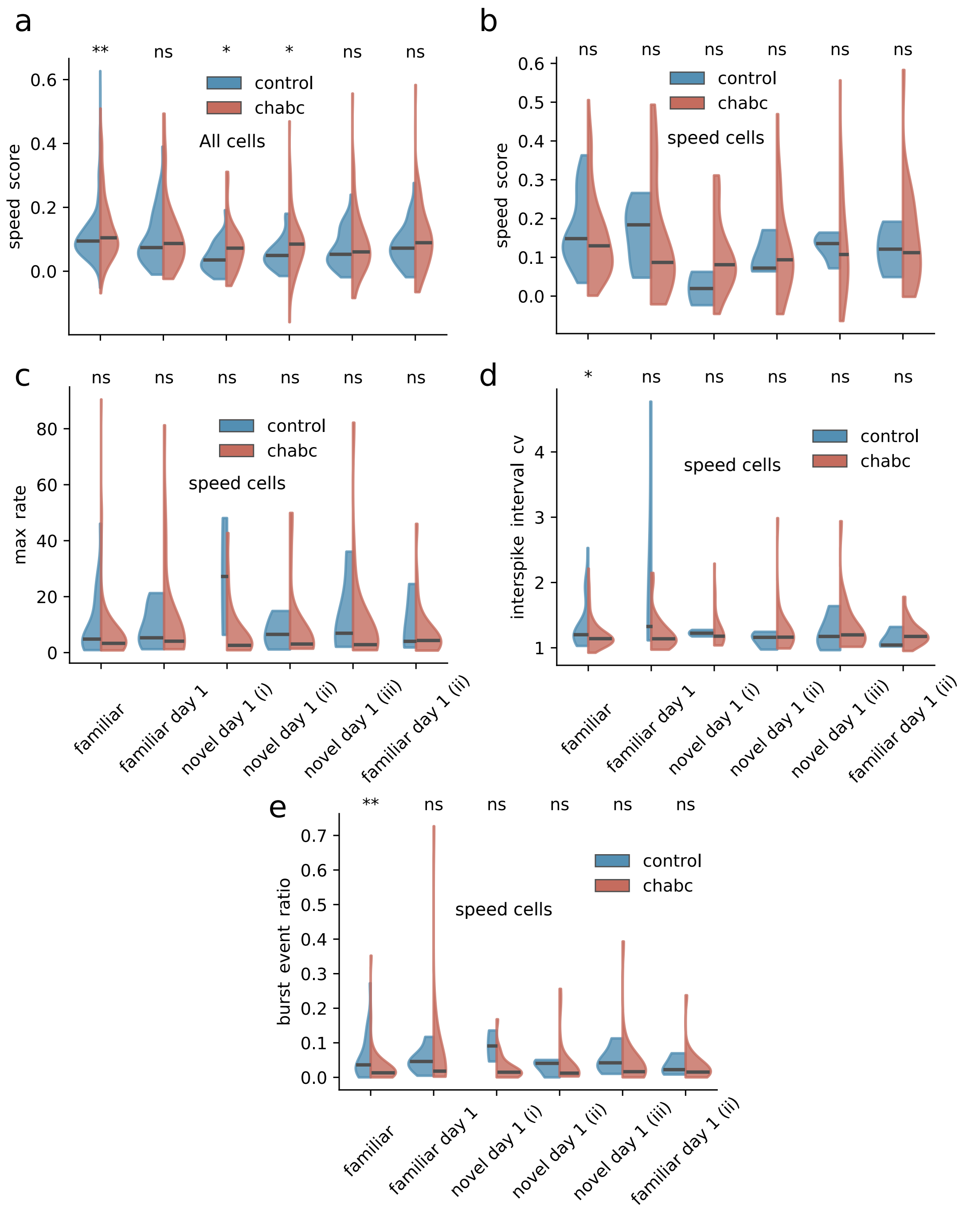

Supplementary Figure 6: Speed modulation. **a**) Speed score for all recorded units that showed speed score above the 95th percentile of shuffled spikes in familiar and novel environment. Median speed score is significantly higher in chABC treated animals in familiar environment and novel (1 and 2). **b**) Units were classified as "speed cells" by computing the 95th percentile of shuffled spikes and using the following criterions: speed score larger than threshold, gridness less than threshold, head direction score less than threshold information rate less than threshold. Speed cells from the two groups did not differ in speed score **b**) or max rate **c**). The coefficient of variation (CV) of the interspike interval was lower in the ChABC treated group **d**) and the burst events more infrequent **e**) in the familiar environment. Violin plots show min to max and median (black line). Width of graph corresponds to number of samples for each value. Mann-Whitney U test (two-sided). $ns = notsignificant$, $*p < 0.05$, $**p < 0.01$, $***p < 0.001$, $****p < 0.0001$. See Supplementary Table 9 for statistics.

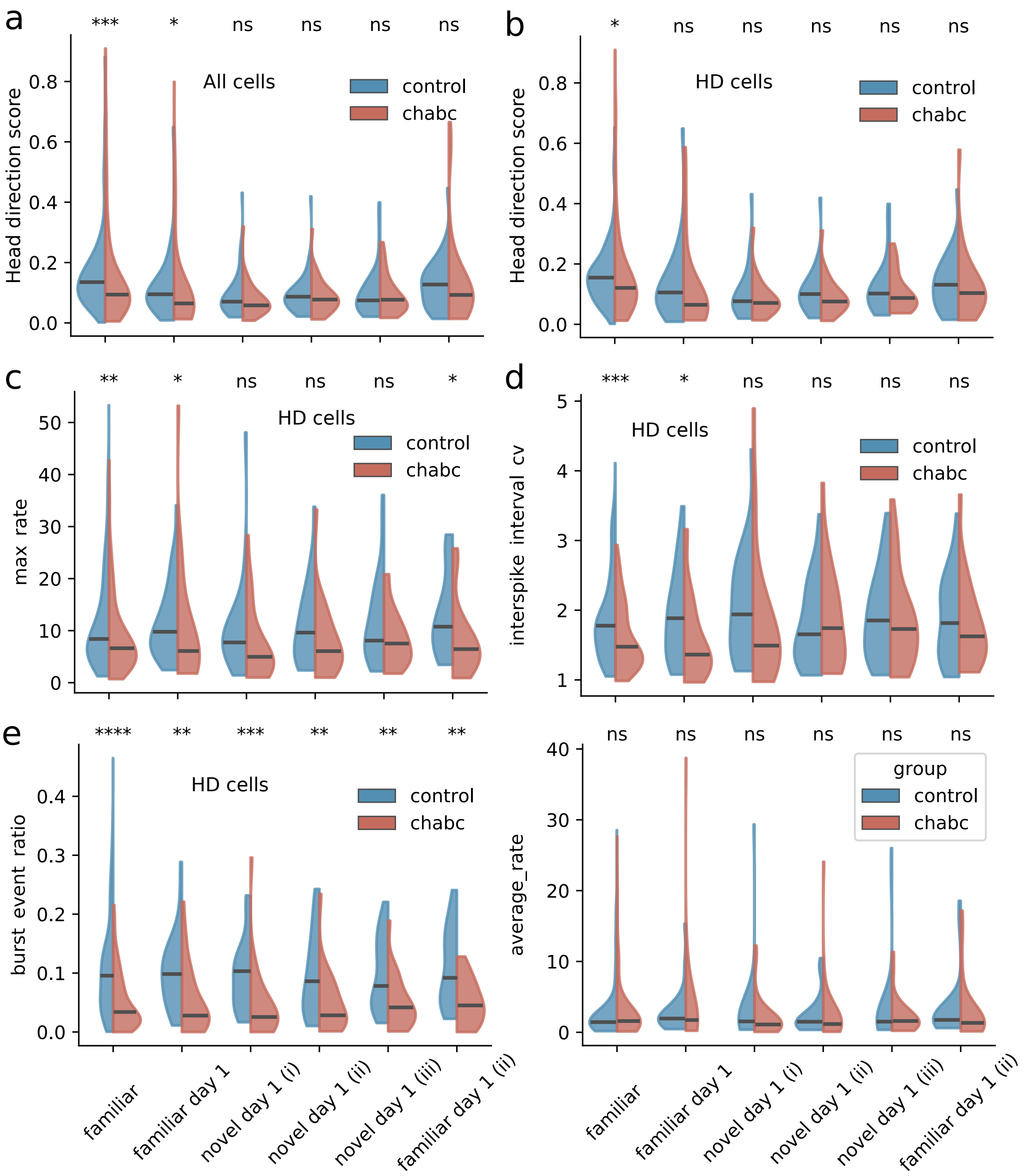

Supplementary Figure 7: Head direction cells show reduced bursting and max rate after PNN removal. **a**) Median head direction score for all units classified as head direction cells (HD cells) was lower in the chABC treated group in the familiar environment. **b**) Units classified as head direction cells but that were not grid cells had lower head direction score after chABC treatment than the control group in familiar environment. **c**) HD cells in the treated group have lower max rate in familiar environments. **d**) HD cells in the treated group have lower interspike interval coefficient of variation (cv) than control. **e**) Burst event ratio was lower in chABC treated animals during all recording sessions. Violin plots show min to max and median (black line). Width of graph corresponds to number of samples for each value. Mann-Whitney U test (two-sided). $ns = notsignificant$, $*p < 0.05$, $**p < 0.01$, $***p < 0.001$, $****p < 0.0001$. See Supplementary Table 10 for statistics.

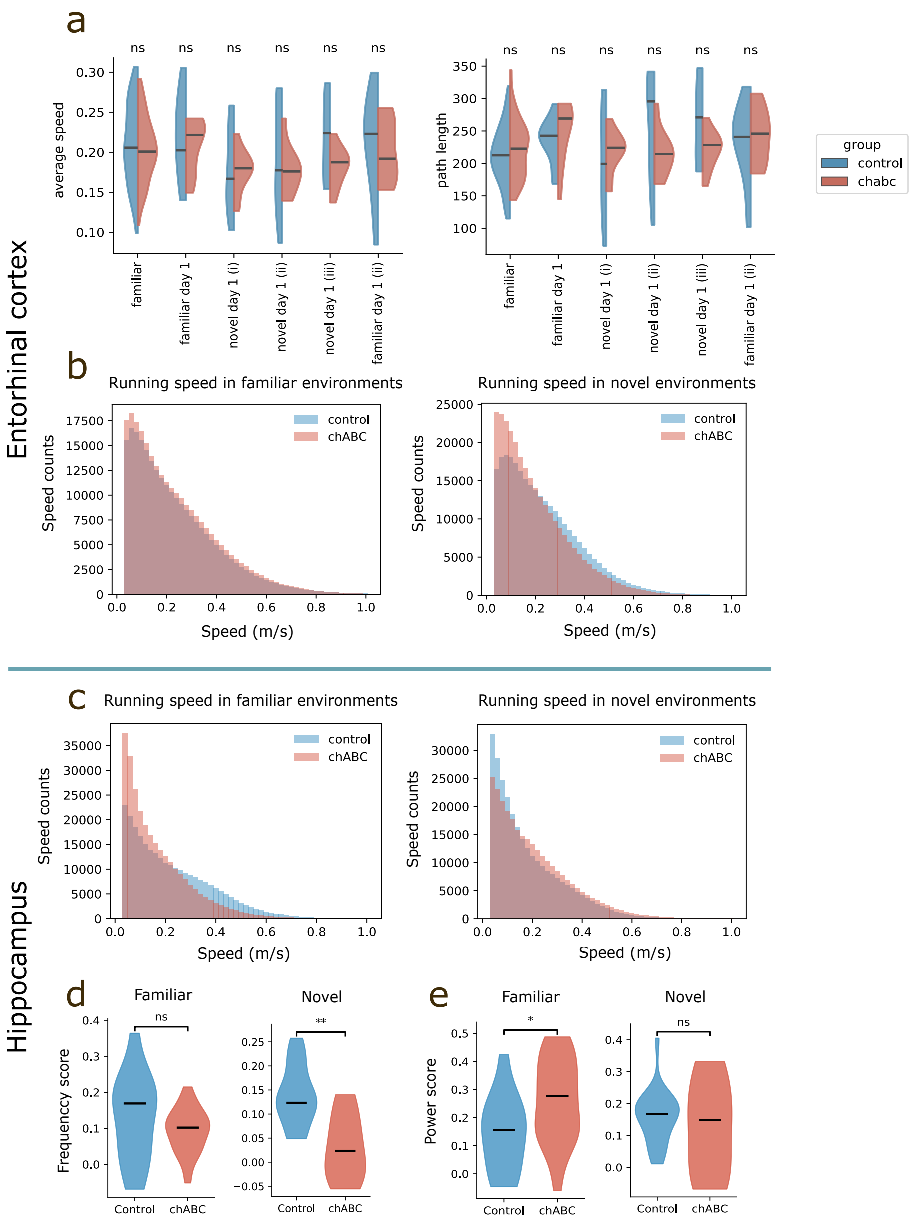

Supplementary Figure 8: Running speed for all animals during exploration of a familiar and novel arena. **a**) Average speed and path length from animals during exploration of familiar and novel environment. We found no significant differences between chABC treated and control animals. b) Running speed from all animals where we recorded local field potential (LFP) in MEC and **c**) hippocampus. **d**) Frequency score (the correlation between instantaneous running speed and theta frequency) from recordings of LFP in hippocampus in familiar and novel environments. **e)** Power score (the correlation between instantaneous running speed and theta power) from recordings of LFP in hippocampus in familiar and novel environments. Violin plot shows min to max and median (black line). Mann-Whitney U test (two-sided). $ns = notsignificant$, $*p < 0.05$, $**p < 0.01$, $***p < 0.001$, $****p < 0.0001$.

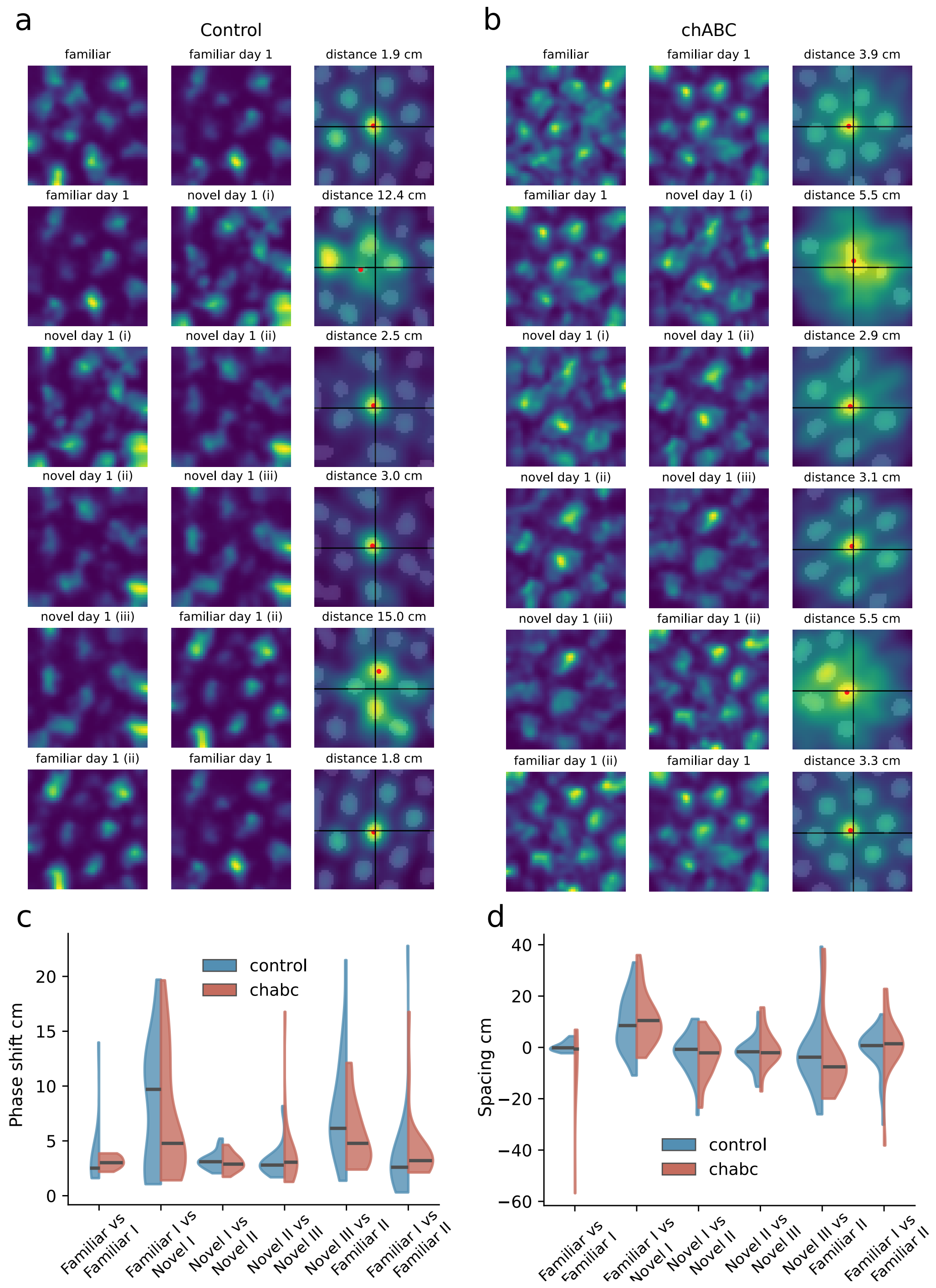

Supplementary Figure 9: Changes in phase and spacing of grid cells a-b) Example of rate maps and cross correlograms from a grid cell in control group (**a**) and chABC treated group (**b**)). Cross correlograms were constructed from two rate maps from the same grid cell in two different environments. **c**) Phase shift was calculated by measuring the distance from origin to the nearest peak in the cross correlogram (marked red). As expected the grid shifts when the animal enters the novel environment (second row), but there is no difference between the two groups. **d**) Changes in spacing for grid cells in different recording sessions. Spacing increase slightly during the first exposure to the novel environment for both groups. Violin plot shows min to max and median (black line).

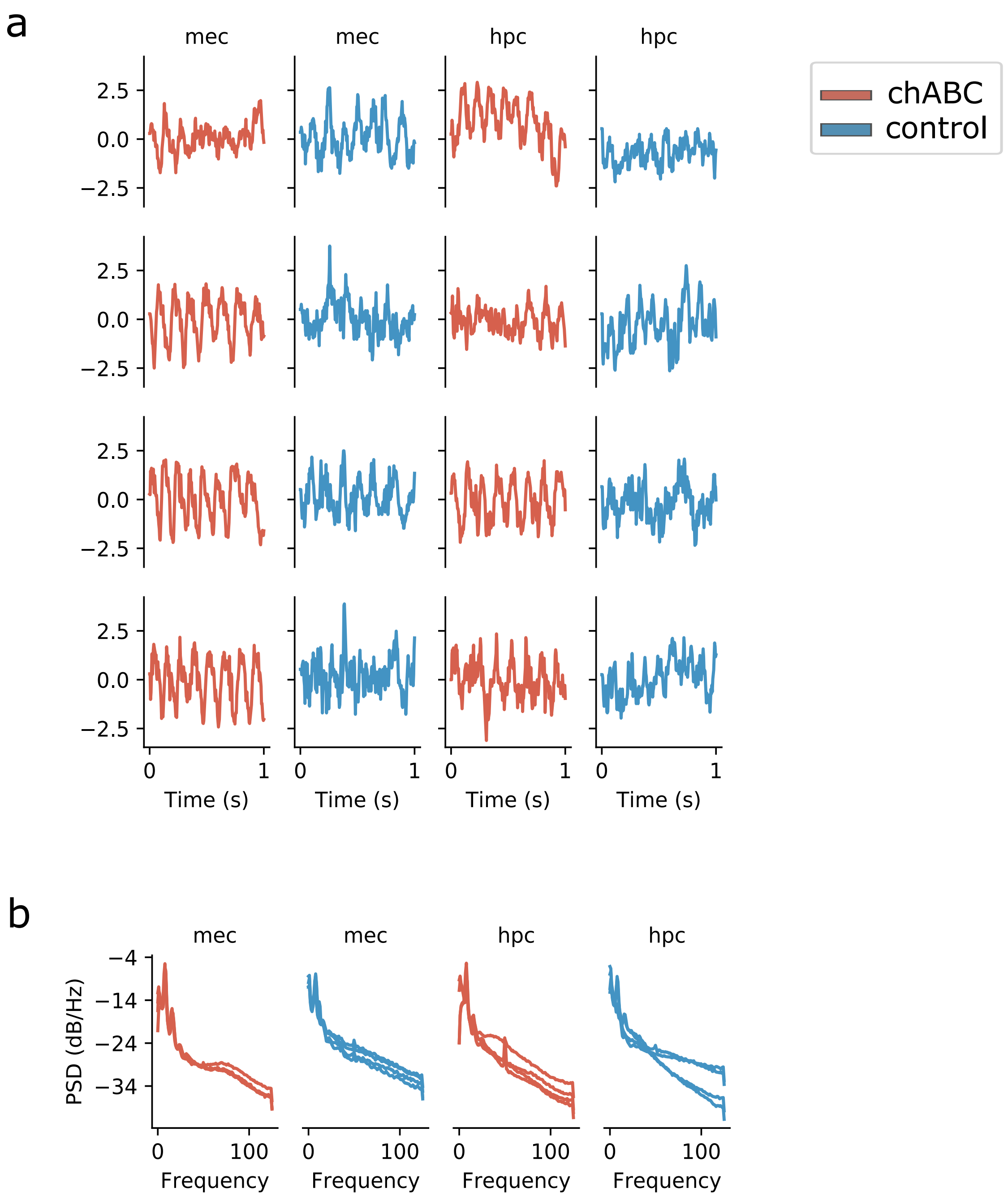

Supplementary Figure 10: Examples of LFP traces from recordings in MEC and hippocampus. **a**) Short traces showing theta oscillations from MEC and hippocampus in chABC treated and control animals. Traces from four different recording sessions for each condition. **b**) Power spectrum density plots from the full recording sessions shown in (a).

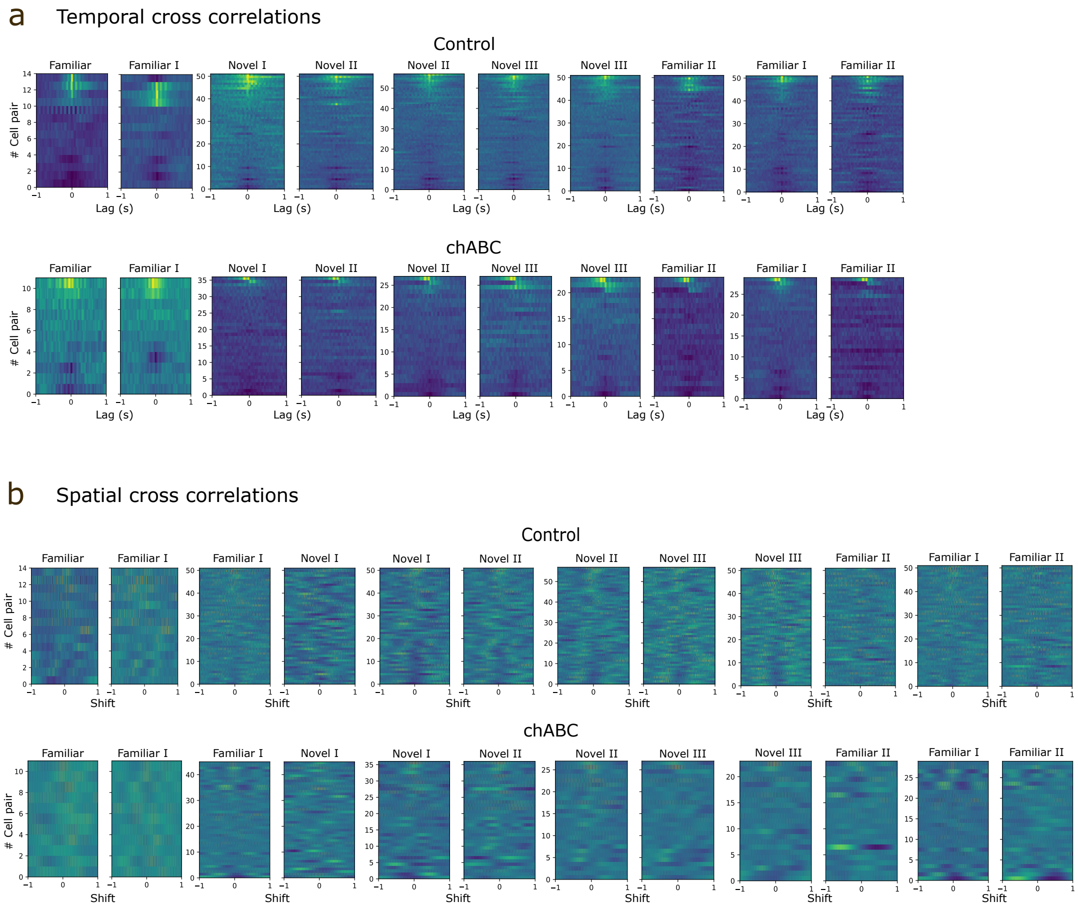

Supplementary Figure 11: Pairwise correlations between grid cells in all environments. **a**) Pairwise temporal correlation histograms of grid cells with brighter color showing z-scored number of co-occuring spikes. Each line represents a pair, sorted by maximum value of central peak, with pair identity maintained across experimental states (left and right panel). **b**) Pairwise spatial correlations (as in **a**, but each line is the flattened spatial cross correlation).

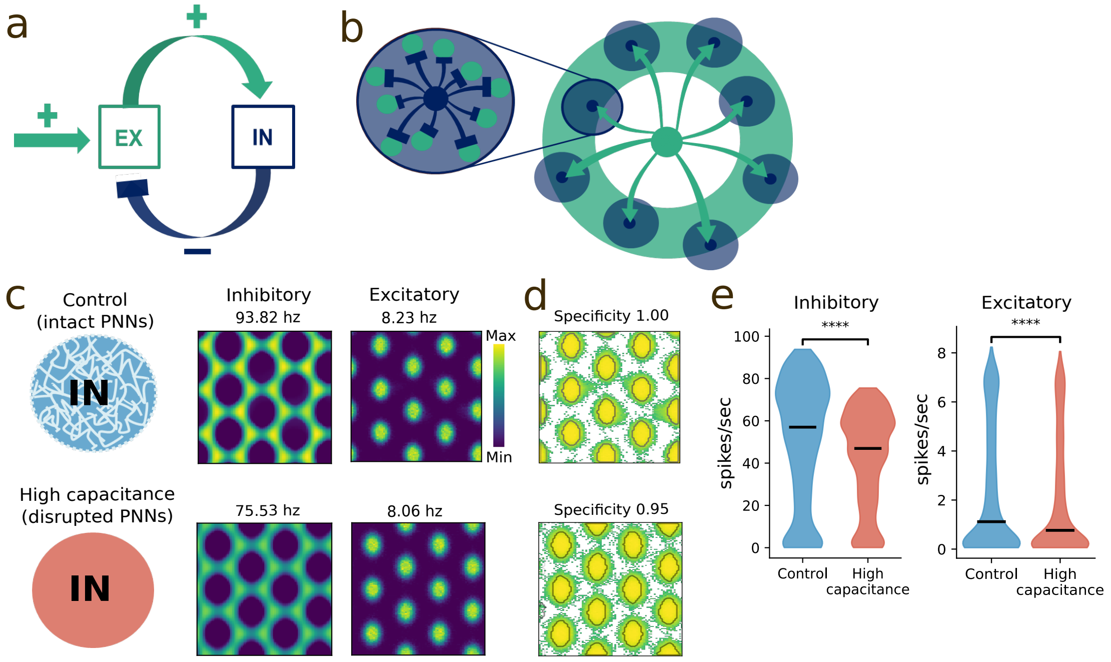

Supplementary Figure 12: Continuous attractor network model with increased capacitance. It has been suggested that PNN removal increase capacitance of the interneurons [**?**], and we tested this by a similar model as used in main **??**). **a**) Schematic outline of the model. Each excitatory neuron (green) is connected to distant inhibitory neurons (dark blue), which in turn inhibit excitatory neurons locally. **b**) A population of excitatory neurons (EX) receive external drive and feedback inhibition by a population of inhibitory neurons (IN). **c**) PNN removal was simulated by increasing the capacitance of the IN population (lower panel). **d**) Grid cells in the increased capacitance network show increased out-of-field firing and thereby lower specificity than control, as seen by the logarithmic color scale. **e**) Average firing rate of both inhibitory and excitatory neurons are lower in the high capacitance network. Thus, increased capacitance in inhibitory neurons affect average firing rate of grid cells similar to the effects seen by reduced inhibition. However, the two models show opposite results in maximum rate. Mann-Whitney U test (two-sided). Violin plot shows min to max and median (large black line). $ns = notsignificant$, $^{*}p < 0.05$, $^{**}p < 0.01$, $^{***}p < 0.001$, $^{****}p < 0.0001$.

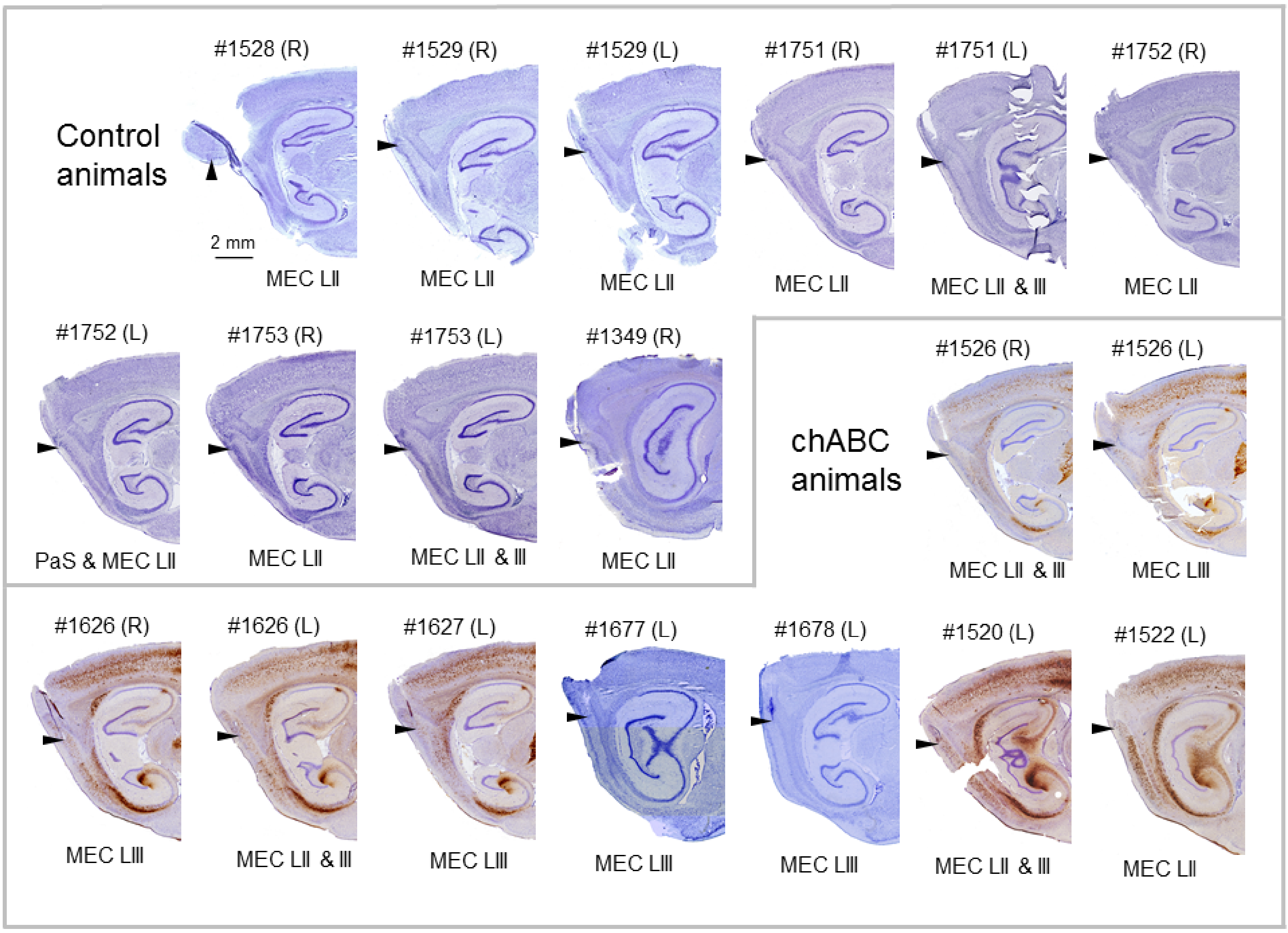

Supplementary Figure 13: Tetrode tracks in MEC from experimental animals. Sections were stained for Nissl bodies to visualize the displacement of cell somas, and WFA labeled with an avidin-streptavidin - 3,3 diaminobenzidine reaction to visualize the area with PNNs removed. Arrows indicate the end of tetrode tracks, numbers indicate animal number, and (R) and (L) indicate the right/left hemisphere with the brain seen from above in the anterior-posterior direction.

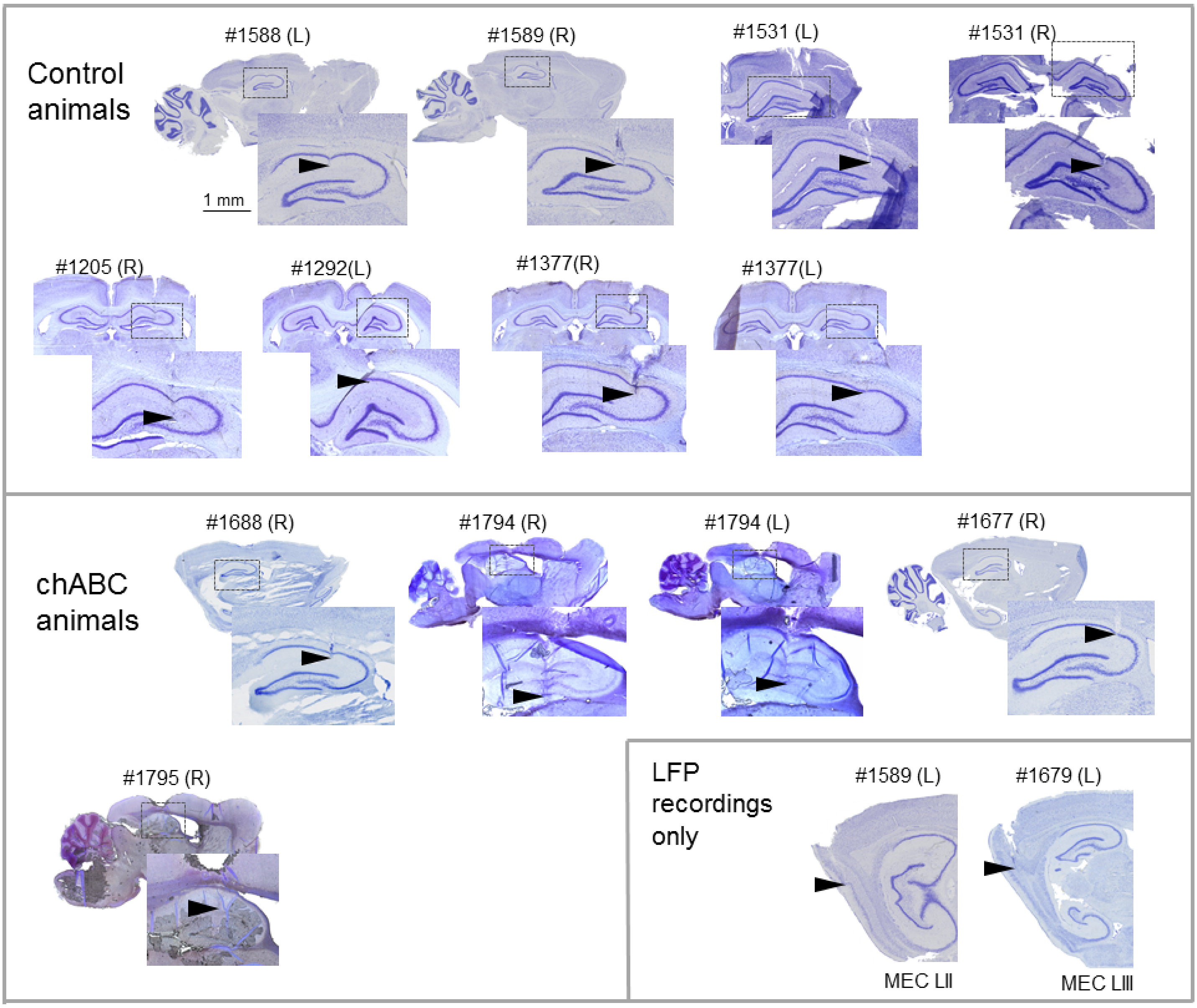

Supplementary Figure 14: Tetrode tracks in hippocampus from experimental animals. Sections were stained for Nissl bodies to visualize the displacement of cell somas. Arrows indicate the end of tetrode tracks, numbers indicate animal number, and (R) and (L) indicate the hemisphere with the brain seen from above in the anterior-posterior direction. Lower right inset shows tetrode tracks in MEC from the left hemisphere of two control animals where we included animals in analysis of LFP recordings but did not record any units.

	Control (n)	chABC (n)	MWU (U, p)	PRS (diff, p)
Average rate	1.53 (86)	1.67 (63)	2695, 0.959	0.13, 0.616
Gridness	0.63 (86)	0.56 (63)	2812, 0.694	0.07, 0.368
Max rate	9.32 (86)	6.93 (63)	3385, 0.009	2.39, 0.012
Information rate	0.36 (86)	0.20 (63)	3688, <0.001	0.17, 0.003
CV of interspike interval	0.99 (86)	0.82 (61)	3520, <0.001	0.17, <0.001
In-field mean rate	2.95 (86)	2.85 (63)	3059, 0.179	0.10, 0.494
Out-field mean rate	0.85 (86)	0.95 (63)	2395, 0.228	0.10, 0.199
Burst event ratio	0.08 (86)	0.02 (63)	4148.50, <0.001	0.06, <0.001
Specificity	0.57 (86)	0.45 (63)	3796, <0.001	0.13, <0.001

Supplementary Table 2: Firing properties of grid cells in a familiar environment for control and chABC groups. Values show median, (number of cells), Two-sided Mann Whitney U test (U, p) and Permutation resampling (PRS) (diff, p).

Between groups	Control (n)	chABC (n)	MWU (U, p)	PRS (diff, p)
Gridness score	0.65 (35)	0.44 (24)	606, 0.004	0.21, 0.005
Spatial stability Familiar I	0.67 (16)	0.65 (16)	139, 0.692	0.02, 0.639
Spatial stability Familiar II	0.72 (16)	0.53 (16)	191, 0.018	0.19, 0.016
Familiar I vs Familiar II	0.63 ± 0.08 (16)	0.57 ± 0.08 (16)	152, 0.376	0.08, 0.162
Novel day1 vs Novel day2	0.76 (12)	0.41 (11)	113, 0.004	0.35, 0.005
Novel day2 vs day3	0.7 (12)	0.38 (5)	52, 0.023	0.32, 0.052
Within groups	Control	chABC		
Spatial stability Familiar I	0.67 (16)	0.65 (16)		
Spatial stability Novel I	0.54 (16)	0.45 (16)		
MWU	174, 0.086	193, 0.015		
PRS	0.13, 0.070	0.21, 0.017		

Supplementary Table 3: Spatial stability of grid cell representations. Upper panel comparison of chABC and control group. Lower panel shows in-group comparisons. The spatial stability of the chABC group is significantly lower in novel I vs familiar I. Values are median, (number of cells), Two-sided Mann-Whitney U test (MWU) (U, p) and Permutation Resampling (PRS) (diff, p).

	Control (n)	chABC (n)	MWU (U, p)	PRS (diff, p)
Power familiar	-6.12 (148)	-4.18 (75)	2832, <0.001	1.94, <0.001
Power novel	-7.17 (33)	-4.8 (24)	126, <0.001	2.37, <0.001
Frequency familiar	8.39 (148)	8.27 (75)	6356, 0.075	0.12, 0.003
Frequency novel	8.39 (33)	8.09 (24)	558, 0.009	0.30, 0.033
Power score familiar	0.14 (148)	0.31 (75)	2800, <0.001	0.17, <0.001
Power score novel	0.10 (33)	0.27 (24)	102, <0.001	0.17, <0.001
Frequency score familiar	0.14 (148)	0.20 (75)	3100, <0.001	0.06, <0.001
Frequency score novel	0.12 (33)	0.19 (24)	200, 0.002	0.07, <0.001

Supplementary Table 4: Theta frequency and power in MEC in familiar and novel environments. Correlation between running speed and power or frequency: power score and frequency score. Median, (n: number of recordings), Two-sided Mann-Whitney U test (MWU) (U, p) and Permutation Resampling (PRS) (diff, p). Number of animals: N=7 for both control and chABC.

	Control	chABC	MWU	PRS
familiar vs familiar day 1	0.57 (14)	0.15 (11)	48 , 0.119	0.42, 0.029
familiar day 1 vs novel day 1 (i)	0.36 (51)	0.21 (45)	874 , 0.045	0.15, 0.023
novel day 1 (i) vs novel day 1 (ii)	0.33 (51)	0.19 (36)	756 , 0.164	0.14, 0.174
novel day 1 (ii) vs novel day 1 (iii)	0.47 (57)	0.31 (27)	579 , 0.069	0.16, 0.126
novel day 1 (iii) vs familiar day 1 (ii)	0.30 (51)	0.25 (23)	586 , 1.000	0.05, 0.603
familiar day 1 vs familiar day 1 (ii)	0.52 (51)	0.20 (29)	529 , 0.036	0.32, 0.018

Supplementary Table 5: Statistics from pairwise temporal cross correlation see **??** and Supplementary Fig. 11. Median, (number of cell pairs), Two-sided Mann-Whitney U test (MWU) (U, p) and Permutation Resampling (PRS) (diff, p).

		Control	chABC	MWU	PRS
stability	familiar vs familiar day 1	0.87 (14)	0.64 (11)	57 , 0.286	0.23, 0.377
	familiar day 1 vs novel day 1 (i)	0.10 (51)	0.05 (45)	800 , 0.011	0.05, 0.139
	novel day 1 (i) vs novel day 1 (ii)	0.78 (51)	0.60 (36)	620 , 0.010	0.18, 0.002
	novel day 1 (ii) vs novel day 1 (iii)	0.83 (57)	0.52 (27)	344 , <0.001	0.30, <0.001
	novel day 1 (iii) vs familiar day 1 (ii)	0.07 (51)	0.01 (23)	465 , 0.158	0.06, 0.284
	familiar day 1 vs familiar day 1 (ii)	0.63 (51)	0.31 (29)	555 , 0.066	0.32, 0.070

Supplementary Table 6: Statistics from pairwise spatial cross correlations. Median, (n), Two-sided Mann-Whitney U test (MWU) (U, p) and Permutation Resampling (PRS) (diff, p).

	Control	chABC	MWU (U,p)	PRS
Average rate	0.43 (52)	0.53 (113)	2454, 0.090	0.11, 0.218
Max rate	6.15 (52)	5.32 (113)	3371, 0.129	0.82, 0.292
Information rate	0.55 (52)	0.43 (113)	3387, 0.116	0.11, 0.134
Interspike interval cv	2.21 (52)	2.19 (113)	2996, 0.840	0.02, 0.910
In-field mean rate	2.97 (43)	2.03 (100)	2813, 0.004	0.94, 0.007
Out-field mean rate	0.19 (52)	0.34 (113)	2117, 0.004	0.15, 0.010
Burst event ratio	0.11 (52)	0.14 (113)	2413.50, 0.066	0.03, 0.155
Specificity	1.04 (43)	0.76 (100)	3051, <0.001	0.28, <0.001
Speed score	0.03 (52)	0.02 (113)	3128, 0.506	0.01, 0.825
Fields area mean	1105.08 (52)	1044.18 (113)	3812, 0.002	60.90, 0.010

Supplementary Table 7: Firing properties of place cells in a familiar arena for control and chABC groups. Values show median, (n), Two-sided Mann-Whitney U test (U, p) test and Permutation resampling (PRS) (diff, p).

Time	Diff	95% CI
0-5 min	0.06	0.13 to 0.25, p = 0.975
5-10 min	0.17	0.07 to 0.40, p = 0.343
10-15 min	0.28	0.08 to 0.48, p = 0.002
15-20 min	0.28	0.09 to 0.48, p = 0.002
20-25 min	0.21	0.02 to 0.41, p = 0.030
25-30 min	0.24	0.05 to 0.43, p = 0.008
30-35 min	0.28	0.08 to 0.48, p = 0.003
35-40 min	0.28	0.09 to 0.48, p = 0.002

Supplementary Table 8: Šidák's multiple comparisons post hoc test for spatial correlations of grid cells in a novel environment. Mean difference between control and chABC (diff) and 95 % CI of diff) .

		Control	chABC	MWU	PRS
max rate	familiar	4.84 (28)	3.32 (83)	913 , 0.092	1.52, 0.163
interspike interval cv	familiar	1.20 (28)	1.14 (83)	824 , 0.022	0.06, 0.054
burst event ratio	familiar	0.04 (28)	0.01 (83)	722 , 0.003	0.02, 0.001
speed score	familiar	0.15 (28)	0.13 (83)	961 , 0.173	0.02, 0.520
speed score all	familiar	0.09 (323)	0.10 (300)	54297 , 0.009	0.01, 0.028

Supplementary Table 9: Statistics for speed modulation. Units without spatial tuning curves (i.e. grid cells, head direction cells and border cells) and speed score > the 95th percentile of shuffled spikes in familiar and novel environment were classified as "speed cells". Note that the overall speed modulation was slightly stronger in the chABC group. Median, (number of cells), Two-sided Mann-Whitney U test (MWU) (U, p) and Permutation Resampling (PRS) (diff, p).

		Control	chABC	MWU	PRS
max rate	familiar	8.40 (139)	6.62 (122)	6754 , 0.005	1.78, 0.022
	familiar day 1	9.78 (28)	6.09 (26)	250 , 0.049	3.70, 0.065
	novel day 1 (i)	7.74 (22)	4.97 (25)	184 , 0.054	2.77, 0.081
	novel day 1 (ii)	9.62 (25)	6.06 (24)	216 , 0.095	3.56, 0.167
	novel day 1 (iii)	8.08 (25)	7.52 (24)	243 , 0.258	0.56, 0.773
	familiar day 1 (ii)	10.76 (24)	6.45 (22)	161 , 0.024	4.31, 0.057
interspike interval cv	familiar	1.78 (139)	1.48 (122)	6348 , <0.001	0.30, <0.001
	familiar day 1	1.88 (28)	1.36 (26)	238 , 0.030	0.52, 0.043
	novel day 1 (i)	1.94 (22)	1.49 (25)	199 , 0.107	0.45, 0.162
	novel day 1 (ii)	1.65 (25)	1.74 (24)	300 , 0.992	0.09, 0.782
	novel day 1 (iii)	1.85 (25)	1.73 (24)	272 , 0.582	0.12, 0.598
	familiar day 1 (ii)	1.82 (24)	1.62 (22)	232 , 0.489	0.19, 0.528
burst event ratio	familiar	0.10 (139)	0.03 (122)	4390, <0.001	0.06, <0.001
	familiar day 1	0.10 (28)	0.03 (26)	185 , 0.002	0.07, 0.002
	novel day 1 (i)	0.10 (22)	0.03 (25)	114 , 0.001	0.08, <0.001
	novel day 1 (ii)	0.09 (25)	0.03 (24)	159 , 0.005	0.06, 0.007
	novel day 1 (iii)	0.08 (25)	0.04 (24)	145 , 0.002	0.04, 0.040
	familiar day 1 (ii)	0.09 (24)	0.05 (22)	132 , 0.004	0.05, 0.013
HD score	familiar	0.15 (139)	0.12 (122)	7282 , 0.049	0.03, 0.009
	familiar day 1	0.11 (28)	0.06 (26)	291 , 0.209	0.04, 0.058
	novel day 1 (i)	0.08 (22)	0.07 (25)	252 , 0.631	0.01, 0.780
	novel day 1 (ii)	0.10 (25)	0.08 (24)	249 , 0.312	0.02, 0.116
	novel day 1 (iii)	0.10 (25)	0.09 (24)	280 , 0.697	0.01, 0.434
	familiar day 1 (ii)	0.13 (24)	0.10 (22)	239 , 0.590	0.03, 0.397
HD score all	familiar	0.14 (258)	0.09 (271)	28910 , 0.001	0.04, <0.001
	familiar day 1	0.09 (45)	0.06 (45)	739 , 0.028	0.03, 0.011
	novel day 1 (i)	0.07 (35)	0.06 (40)	589 , 0.241	0.01, 0.607
	novel day 1 (ii)	0.09 (40)	0.08 (35)	643 , 0.548	0.01, 0.474
	novel day 1 (iii)	0.07 (37)	0.08 (34)	608 , 0.813	0.00, 0.856
	familiar day 1 (ii)	0.13 (35)	0.09 (36)	550 , 0.361	0.03, 0.194

Supplementary Table 10: Statistics for head direction cells. Median, (number of cells), Two-sided Mann-Whitney U test (MWU) (U, p) and Permutation Resampling (PRS) (diff, p).

	Excitatory	Control Inhibitory	Case Inhibitory	Unit
C_m	70.0	30.0	30.0	pF
$C_m(supplementary\ experiment)$	70.0	30.0	40.0	pF
Δ_T	2.5	2.5	2.5	ms
E_L	-60.0	-60.0	-60.0	mV
$E_{rev,AMPA}$		0.0	0.0	mV
$E_{rev,NMDA}$		0.0	0.0	mV
$E_{rev,GABA_A}$	-75.0			mV
$\bar{g}_{AMPA}$		0.02	0.005	nS
$\bar{g}_{NMDA}$		0.007	0.0065	nS
$\bar{g}_{GABA_A}$	0.01			nS
$\tau_{rise,AMPA}$		1.0	1.0	ms
$\tau_{rise,NMDA}$		10.0	10.0	ms
$\tau_{rise,GABA_A}$	1.0			ms
$\tau_{decay,AMPA}$		5.0	5.0	ms
$\tau_{decay,NMDA}$		100.0	100.0	ms
$\tau_{decay,GABA_A}$	5.0			ms
N	10000	10000	10000	
V_m	-60.0	-60.0	-60.0	mV
V_{peak}	-40.0	-40.0	-40.0	mV
V_{reset}	-60.0	-60.0	-60.0	mV
V_{th}	-50.0	-45.0	-45.0	mV
g_L	4.0	4.5	4.5	nS
r_{inner}	0.2π	0	0	
r_{outer}	0.4π	0.15π	0.15π	
$p_{\bar{g}}$	0.1	0	0	nS
p_{rate}	850.0	0.0	0.0	Hz
t_{ref}	1.0	1.0	1.0	ms

Supplementary Table 11: Parameters used in the can model.
