# Supplementary material for: Perineuronal nets stabilize the grid cell network
Source: Nat Commun. 2021 Jan 11;12:253. doi: 10.1038/s41467-020-20241-w (PMC7801665; doi:10.1038/s41467-020-20241-w)
Supplement: Supplementary file 3 — Reporting Summary [file 41467_2020_20241_MOESM3_ESM.pdf]

nature research

Corresponding author(s): Torkel Hafting

Last updated by author(s): Oct 17, 2020

Reporting Summary

Nature Research wishes to improve the reproducibility of the work that we publish. This form provides structure for consistency and transparency in reporting. For further information on Nature Research policies, see our Editorial Policies and the Editorial Policy Checklist.

Statistics

For all statistical analyses, confirm that the following items are present in the figure legend, table legend, main text, or Methods section.

n/a	Confirmed	
☐	☒	The exact sample size (*n*) for each experimental group/condition, given as a discrete number and unit of measurement
☐	☒	A statement on whether measurements were taken from distinct samples or whether the same sample was measured repeatedly
☐	☒	The statistical test(s) used AND whether they are one- or two-sided<br>*Only common tests should be described solely by name; describe more complex techniques in the Methods section.*
☐	☒	A description of all covariates tested
☐	☒	A description of any assumptions or corrections, such as tests of normality and adjustment for multiple comparisons
☐	☒	A full description of the statistical parameters including central tendency (e.g. means) or other basic estimates (e.g. regression coefficient) AND variation (e.g. standard deviation) or associated estimates of uncertainty (e.g. confidence intervals)
☐	☒	For null hypothesis testing, the test statistic (e.g. *F*, *t*, *r*) with confidence intervals, effect sizes, degrees of freedom and *P* value noted<br>*Give P values as exact values whenever suitable.*
☐	☒	For Bayesian analysis, information on the choice of priors and Markov chain Monte Carlo settings
☐	☒	For hierarchical and complex designs, identification of the appropriate level for tests and full reporting of outcomes
☐	☒	Estimates of effect sizes (e.g. Cohen's *d*, Pearson's *r*), indicating how they were calculated

Our web collection on statistics for biologists contains articles on many of the points above.

Software and code

Policy information about availability of computer code

Data collection	Electrophysiological data was collected using daqUSB from Axona (Herts, UK). Spike sorting was done offline using the software Tint from Axona. Images of histological sections were acquired using Fusion Software (Bitplane) and Axiovision (Carl Zeiss).
Data analysis	Data was analysed using Python 3.6. Custom made code is avaliable at https://github.com/CINPLA/pnn-mec and requirements described within. Version: 669f8270bc2f32b534dd599c5fb5101daba9a8c9

For manuscripts utilizing custom algorithms or software that are central to the research but not yet described in published literature, software must be made available to editors and reviewers. We strongly encourage code deposition in a community repository (e.g. GitHub). See the Nature Research guidelines for submitting code & software for further information.

Data

Policy information about availability of data

All manuscripts must include a data availability statement. This statement should provide the following information, where applicable:

- Accession codes, unique identifiers, or web links for publicly available datasets
- A list of figures that have associated raw data
- A description of any restrictions on data availability

All data are available in the excel file SourceData.xlsx.
The source code used for analysis will be made available at https://github.com/CINPLA/pnn-mec.
Raw data will be made available at https://gitea.expipe.sigma2.no/anecc/pnn-mec.

Field-specific reporting

Please select the one below that is the best fit for your research. If you are not sure, read the appropriate sections before making your selection.

- [x] Life sciences
- [ ] Behavioural & social sciences
- [ ] Ecological, evolutionary & environmental sciences

For a reference copy of the document with all sections, see nature.com/documents/nr-reporting-summary-flat.pdf

Life sciences study design

All studies must disclose on these points even when the disclosure is negative.

Sample size	No sample size calculation was perfomed.
Data exclusions	Animals were excluded from the study if enzymatic injections to remove PNNs could not be visualized or did not cover parts of MEC after immunostaining. No analysis were performed with these animals. Single units were excluded in cases where the autocorrelation of spikes showed spiking within the 2-3 ms refractory period. If further spike sorting could not remove these, the unit was discarded from analysis.
Replication	To enable reproducibility, a protocol for enzymatic injections and electrophysiological data collection was compiled prior to the beginning of this project. This was done by a separate experimenter as part of a master thesis. During data collection for this paper, the same protocol was followed for all animals. Since data collection was performed over a longer period of time, we performed preliminary analyses that was later reconfirmed after adding more experiments. Downsampling of the data yielded similar results.
Randomization	Data were collected over a period of two years. At first animals from the same litter were randomly used as controls or injected with chABC. Based on the successful data collection and enzymatic treatment in the initial round of experiments, further experiments were performed to increase sample size for each group, with the intention of having similar sample sizes.
Blinding	Data collection was not blinded. Surgery, electrophysiological recordings and offline spike sorting was performed by the same experimenter. After experiments ended and successful enzymatic degradation of PNNs confirmed, animals were included in the experimental group. All analysis and statistics were performed after data collection was completed, by people not involved in data collection.

Reporting for specific materials, systems and methods

We require information from authors about some types of materials, experimental systems and methods used in many studies. Here, indicate whether each material, system or method listed is relevant to your study. If you are not sure if a list item applies to your research, read the appropriate section before selecting a response.

Materials & experimental systems

n/a	Involved in the study	
[ ]	[x]	Antibodies
[x]	[ ]	Eukaryotic cell lines
[x]	[ ]	Palaeontology and archaeology
[ ]	[x]	Animals and other organisms
[x]	[ ]	Human research participants
[x]	[ ]	Clinical data
[x]	[ ]	Dual use research of concern

Methods

n/a	Involved in the study	
[x]	[ ]	ChIP-seq
[x]	[ ]	Flow cytometry
[x]	[ ]	MRI-based neuroimaging

Antibodies

Antibodies used	Primary antibodies:<br>Wisteria floribunda agglutinin (WFA): #L1516, Sigma-Aldrich. Chondroitin-6 sulfated stubs: MAB 2035, Millipore. Goat anti-PV, #PVG-214, Swant. Rabbit-anti vGlut1, rabbit anti-vGlut2 or rabbit anti-vGat: synaptic vesicle antibodies kindly donated by Dr. Farrukh Chaudry, UiO.<br><br>Secondary antibodies:<br>Streptavidin Alexa 488: #S-11223, Life. Donkey-anti mouse Alexa 594: #A-21203, Life. Donkey anti-goat Alexa 594: #A-11058, Life. Streptavidin Alexa 647: #S-21374, Life. Chicken anti-rabbit Alexa 488: #A-21441, Life.<br><br>For DAB staining: ABC Peroxidase Standard Staining Kit, Thermo Fisher Scientific. Staining was visualized by adding a 3,3-diamonobenzidine hydrochloride solution, Sigma-Aldrich Chemie.
Validation	The lectin Wisteria floribunda agglutinin (WFA) has shown preferential reactivity with glycans containing terminal N-acetylgalactosamine (GalNAc) residues (Härtig & Brauer, 1992), and is has been routinely used to visualize PNNs in mammals

(Fawcett et al., 2019).
Chondroitin-6 sulfated stubs, MAB2030: This antibody only reacts with digested material. Fixed tissue must be digested with chondroitinase ABC (Sigma). Reine, T. M., Grøndahl, F., Jenssen, T. G., Hadler-Olsen, E., Prydz, K., & Kolset, S. O. (2013). Reduced sulfation of chondroitin sulfate but not heparan sulfate in kidneys of diabetic db/db mice. Journal of Histochemistry & Cytochemistry, 61(8), 606-616.
Goat anti-PV, PVG-214: Documentation provided by the manufacturer: https://www.labome.com/product/SWant/PVG-214.html

Animals and other organisms

Policy information about studies involving animals; ARRIVE guidelines recommended for reporting animal research

Laboratory animals	All animals used were rattus norvegicus, Long-Evans, male rats, age 3-8 months purchased from Janvier and/or bred locally.
Wild animals	*Provide details on animals observed in or captured in the field; report species, sex and age where possible. Describe how animals were caught and transported and what happened to captive animals after the study (if killed, explain why and describe method; if released, say where and when) OR state that the study did not involve wild animals.*
Field-collected samples	*For laboratory work with field-collected samples, describe all relevant parameters such as housing, maintenance, temperature, photoperiod and end-of-experiment protocol OR state that the study did not involve samples collected from the field.*
Ethics oversight	Experiments were performed in accordance with the Norwegian Animal Welfare Act and the European Convention for the Protection of Vertebrate Animals used for Experimental and Other Scientific Purposes.

Note that full information on the approval of the study protocol must also be provided in the manuscript.
